# Challenges to polycentric governance of an international development project tackling land degradation in Swaziland

**DOI:** 10.1007/s13280-016-0791-8

**Published:** 2016-06-06

**Authors:** Steven E. Orchard, Lindsay C. Stringer

**Affiliations:** Sustainability Research Institute (SRI), School of Earth and Environment, University of Leeds, Leeds, LS2 9JT UK

**Keywords:** Erosion, Soil degradation, Natural resource management, Sustainable land management, Rangelands, Semi-arid

## Abstract

To effectively address the drivers and impacts of land degradation requires polycentric governance systems that facilitate international development projects (IDPs). This paper analyses an IDP aiming to reduce land degradation in Swaziland. A longitudinal-style qualitative approach draws on repeat household surveys, semi-structured interviews and focus groups. We aim to identify the changes that have taken place since the departure of the IDP funders, and the subsequent dynamics between stakeholders. We: (1) chart the evolution of the institutional structures and processes of the IDP; and (2) assess community perceptions of IDP outcomes. Lack of meaningful participation at various stages of the PMC caused the project to lose momentum following the departure of the funders. We discuss these findings in relation to a polycentric approach, and identify how multi-stakeholder IDP can be facilitated as part of wider polycentric governance approaches to inform policies to combat land degradation within Swaziland and more widely.

## Introduction

Land degradation is a global problem (UNCCD 1994). The UNEP (2011) defines land degradation as “…reduction in the capacity of the land to provide ecosystem goods and services and assure its functions over a period of time for its beneficiaries” UNEP (2011, p. 1). Estimates of total degraded area vary from just less than 10 % to approximately 40 % of the Earth’s 148 300 000 km^2^ land surface (Gibbs and Salmon 2015). Efforts to prevent, reduce and rehabilitate degraded areas are enshrined in international policies and development frameworks, including the sustainable development goals (SDGs). Major causes of land degradation include: (1) human activities resulting in unsustainable land use and management, such as deforestation, overexploitation of natural resources and overgrazing; and (2) biophysical factors such as topography, soil quality and climate change and variability (Kairis et al. 2014). Consequences include: reduced productivity, food insecurity, biodiversity loss and loss of ecosystem goods and services (UNEP 2011), as well as knock on impacts for human health, livelihoods and wellbeing. These causes and consequences occur over multiple interacting temporal and spatial scales (see Reynolds et al. 2007). Land degradation is therefore a complex, uncertain and multi-scale phenomenon, affecting multiple actors and agencies, and requiring transparent decision making that is responsive to changing circumstances (Stringer et al. 2009). There is growing acknowledgement that centralised, top-down mechanisms are inadequate for tackling land degradation as well as ensuring the sustainable use of natural resources more widely (Nagendra and Ostrom 2012). This has led to a shift in design and implementation of international development projects (IDPs) towards decentralisation and community participation (Stringer et al. 2014).

### Central concepts

Participation often takes place in IDP that form part of a broader programme approach. IDP adopts participatory approaches in an attempt to foster institutional mechanisms through which local stakeholders’ needs and interests can theoretically be included in the design and implementation of natural resource management (Akbulut and Soylu 2012). Indeed, international agencies such as the United Nations (UN) and World Bank (WB) advocate community participation within their IDP for building effective, efficient and equitable natural resource governance. However, IDP typically faces time, cost and quality constraints, making them largely social and political undertakings (Bixler 2014): social in that they aim to improve the wellbeing of target populations; political in that the choice of issue, location and target group are decisions made by IDP donors, agencies, political leaders and policy makers (Diallo and Thuillier 2004). IDP implementation also typically occurs at the local level, in specific locations and by particular groups of people (Nagendra and Ostrom 2012). Whilst IDP can facilitate sustainable development if designed, implemented and managed appropriately (Ika 2012), they can exacerbate land degradation challenges if not (MEA 2005). Ensuring the wellbeing of populations faced with the challenge of land degradation requires multi-tier governance solutions that facilitate IDP to address both the drivers and impacts of the problem (Nagendra and Ostrom 2012).

Polycentric governance enhances participation by fostering inclusive decision making from divergent groups, between and among multiple centres of authority and scales of governance (Andersson and Ostrom 2008). The types of ecosystem service that natural resources provide, change as the physical scale of the resource changes (Nagendra and Ostrom 2012). For example, soils support the provision of food at the local level, and carbon to regulate the climate at the global level. No single level of governance can provide incentives for users to safeguard the long-term delivery of such a variety of services, while bestowing management of natural resources to external experts is unlikely to be sustainable. The complexity of natural resources at local, regional, national and global levels requires complex governance systems involving input from local resource users in diverse fashions. Polycentric governance can foster the necessary relationships between and among actors who have a stake in the resource at multiple scales. Hence, it is a useful approach for encouraging flexibility, interlinkages, adaptation and resilience into the system through developing structures and processes to match the multi-scale nature of such resources (Ostrom 2005).

**Fig. 1:**
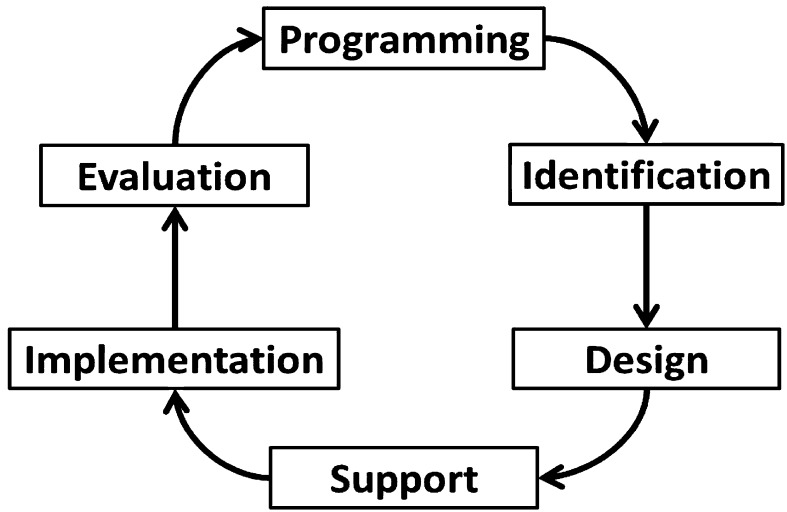
Project management cycle, adapted from Biggs and Smith (2003)

IDP progress in broadly similar ways regardless of the issue being targeted (Crawford and Bryce 2003). Activities typically follow a project management cycle (PMC) as a rational way of conceptualising and managing such projects (Biggs and Smith 2003). Academic analyses focus predominantly on evaluating the success of either project implementation or project outcomes, overlooking aspects pertinent to distinctive phases of the PMC where changes to governance, and therefore opportunities for stakeholder participation, can occur (Khang and Moe 2008). Biggs and Smith (2003) present a framework (Fig. 1) that identifies phases present in almost all project cycles: programming, identification, design, support, implementation and evaluation (Table 1). Learning is integral: i.e. “…making adjustments during the project cycle in response to ongoing events and taking account of past experience in future planning” (Biggs and Smith 2003, p. 1743). Furthermore, the framework considers processes of participation at every stage, and can incorporate a range of assessment criteria at various points in the cycle, e.g. environment, gender, empowerment, capacity building, institutional development and sustainability. Recognising the intangible nature of many IDP results, the framework can be used to assess and forecast project success by analysing the complex relationships between stakeholders, progressively assessing performance at each stage of the PMC to provide insights into the problems and challenges of governance. Hence, a dynamic framework is developed that identifies various success criteria and institutional aspects at different phases that shape IDP success or failure.

**Table 1:** Description of project management cycle phases

Phase	Description
Programming	A broad plan is developed considering the local, national and international context. An overall framework of objectives for a specific country, issue or sector within which single projects can be identified and conducted is agreed upon
Identification	The issues and needs of a particular group are assessed, influenced by pressure from political, social, cultural, ethnic or other groups. Ideas to address these needs are produced and analysed. This may involve consultation with potential beneficiaries through participatory rural appraisal (PRA) techniques
Design	Ideas are worked into tangible operational strategies to be measured against various criteria (e.g. gender, environment, livelihoods, sustainability, etc.) which are contingent on the source and type of support necessary
Support	Numerous types of assistance are sought, e.g. financial, political, institutional, etc. Obtaining the required support involves considerable negotiation and can lead to alterations in the project design
Implementation	Operational strategies are conducted while continuously monitoring progress towards projected objectives. Alterations are frequently made to the original plans in light of unanticipated circumstances and events
Evaluation	Success or failure is evaluated in terms of its impact on stakeholders, achievement of objectives and the lessons learned. Lessons are fed back into the ‘Programming’ phase to inform future planning

IDP is understudied in the PMC literature (Ika et al. 2010), and little is understood about the factors at various stages of the PMC that shape variation of outcomes within decentralised IDP (Khang and Moe 2008), i.e. : the interaction between and among multiple actors, authorities, organisations and sectors at multiple scales of governance that shape IDP success or failure. Furthermore, IDP evaluations often take place immediately after a project without sufficiently considering the longer-term impacts or successes (Ika 2012; Bixler 2014). To address this gap, this paper links the polycentric governance and IDP management literatures, and applies the PMC framework to an IDP aiming to reduce land degradation in Swaziland.

This paper explores an IDP commencing in December 2000 aiming to rehabilitate degraded land. We analyse the situation after the departure of funders in January 2004 building on studies by Stringer (2004) and Stringer et al. (2007). The IDP involved a range of stakeholders from multiple levels, including the Government of Swaziland’s Ministry of Agriculture and Cooperatives, the Japanese International Cooperation Agency (JICA), a Swazi NGO, as well as local people. This paper aims to identify the changes that have taken place since the departure of the IDP funders, and the subsequent dynamics between stakeholders. We: (1) chart the evolution of the institutional structures and processes of the IDP; and (2) assess community perceptions of IDP outcomes. Lack of meaningful participation at various stages of the PMC caused the project to lose momentum following the departure of the funders. We discuss these findings in relation to a polycentric approach, and identify how multi-stakeholder IDP can be facilitated as a part of wider polycentric governance approaches to inform policies to combat land degradation within Swaziland and more widely.

## Materials and methods

### Study country: Swaziland

Three quarters of Swaziland’s 1419 623 inhabitants live in rural areas and typically engage in subsistence agriculture (World Bank 2015). Just over two-thirds of Swaziland’s 17, 364 km^2^ area is under agricultural use for arable crop farming and livestock production (World Bank 2015). Land degradation is a major environmental problem (Tfwala et al. 2012), with just over half the country’s area suffering from some form of land degradation (World Bank 2015). Mismanagement of rangelands through overgrazing, uncontrolled burning and vegetation depletion cause soil erosion and gullying, whilst degradation of cultivated land results from inadequate nutrient replenishment causing a depletion of soil fertility and weed infestations (Stringer et al. 2007; Manyatsi and Maseko 2010).

Swaziland has an unusual land tenure system, with 56 % designated as Swazi National Land (SNL) held in trust for the nation by the King (Mavimbela et al. 2010). Plots for small-scale agriculture are allocated by chiefs to married males (Funnell 1991). Approximately three quarters of the population live on SNL (Xaba and Masuku 2013), where the principal crop is maize, though groundnuts, dry beans, sorghum, pumpkins, jugo beans, soya beans and sweet potatoes are also often grown (Mavimbela et al. 2010). 44 % of land is title deed land (TDL), where exclusive access rights are defined and typically allocated to corporate actors (Mushala et al. 1998). TDL is typically used for commercial purposes and characterised by high levels of investment to grow high values crops (e.g. sugar cane, citrus fruits and trees for timber). This paper focuses on an IDP on SNL.

The monarchy and chiefs dominate the country’s political structure, with their authority legitimised by means of a patriarchal society and control over land, access to cattle and wives. Multiparty politics has been illegal since suspension of the constitution in 1973, with all powers vested in the King since 1979. The current King, Mswati III, ascended to the throne in 1986. Parliament is based on the traditional *Tinkhundla* system, whereby the public vote for candidates from an approved list. This provides the opportunity for the King to distribute royal power throughout the country whilst maintaining central control and averting much of society from participating in political processes. Little real progress has been made towards democratisation, and the alienation of citizens from formal political processes provides an interesting background context against which participatory IDP are superimposed.

### Research design and methodology

Data were collected during May–October 2002 and September–October 2014 (see Stringer (2004) for more details and Table 2 for a summary).

**Table 2:** Data collected during 2002 and 2014

Data collection method	2002	2014
Household questionnaires	74	84
Transect walks with key households	3	3
Semi-structured interviews with key households	3	3
Focus group with original project committee members	1	1
Semi-structured interviews with project workers	46	0
Focus group with JICA consultants	1	0
Series of interviews with original project committee chair	0	1

All households in the study chiefdom were surveyed during both 2002 (*n* = 74) and 2014 (*n* = 84). Questionnaires sought information on local livelihoods, land use practices and environmental priorities, including the types of environmental changes that have occurred in the community over the last (up to) 50 years and how they affected livelihood strategies and household wellbeing. Case study households were selected for transect walks and semi-structured interviews based on survey responses, using purposive sampling (see Stringer 2004). The same case study households participated in 2014 as in 2002, at which time, their involvement in the research was based on them: having access to up to 4 ha of land; having been established in the area over 20 years. Each household represented a different level of wealth and income relative to other households in the chiefdom (see Stringer 2004). Transect walks through arable plots aided familiarisation with local context, history and issues relating to the community, while also enabling more targeted questioning for the subsequent semi-structured interviews. Semi-structured interviews with the same households provided in-depth past and present perspectives on degradation and the project’s structures, processes and impact during the two periods covered by the data collection. They elucidated the involvement of community members at each phase of the PMC, particularly: (a) the design of the project (2002), (b) the implementation of the project (2002 and 2014), (c) the monitoring and evaluation of the project (2014).

Semi-structured interviews with people working on the IDP in 2002 focused on how participatory it was, what it would achieve in terms of social and environmental benefits, and how they (and their household) would gain from involvement. Focus groups with six members of the village-level project committee were conducted towards the end of data collection in both 2002, and four members in 2014, to: (1) explore the degree of consensus between committee members after completion of the implementation phase (2002), and (2) subsequent handing over of the project to the community (2014). Care was taken to ensure effective facilitation throughout the focus groups, with all participants given the opportunity to contribute. No individual was allowed to dominate discussions. Iterative reflections were carried out jointly with participants during both focus groups to determine how and why any conflicts in information may have occurred, and also served to validate the findings from semi-structured interviews with key households. Data were supplemented with a series of short semi-structured interviews in 2014 with the chairman of the original project committee. Consultants working for the project’s international donor, JICA, were interviewed in 2002. However, we were unable to track them down for repeat interviews in 2014. Application of all methods (i.e. transect walks, questionnaires, semi-structured interviews and focus groups) lasted no longer than one and a half hours each, were undertaken in the local language with a translator. Detailed notes were made including translated direct quotations, which were then transferred to electronic copies.

Data analysis was iterative, with qualitative data initially coded under themes relating to different stages of the PMC, then subcategorised according to group/stakeholder. Patterns within codes and subcategories were then identified and grouped to facilitate identification of the structures, processes and impacts that shaped governance of the project. This is consistent with the Grounded Theory approach (Corbin and Strauss 1990), as categories emerged through iterative data analysis, refining the codes as new data were evaluated. This resulted in a cyclical process culminating in inductive interpretation and explanation of results.

## Results

This section sets out the results, drawing on data from both 2002 and 2014.

### Background to the IDP and the situation as of 2002

#### Programming

In 1996, Swaziland’s government requested the government of Japan to investigate the improvement of degraded land in Swaziland’s middleveld region. Consultants conducted literature and policy reviews and highlighted gullying and erosion on communal rangeland as the most pressing form of land degradation. JICA, working with Swaziland’s Ministry of Agriculture and Cooperatives (MOAC), deemed that addressing degradation on communal land would allow decentralised and participatory approaches to be enacted, in line with the dominant international development practice of the time. Such an approach also supported the consultative, participatory approach outlined in the UN Convention to Combat Desertification, the main international treaty for dealing with land degradation, to which Swaziland is a signatory.

#### Identification

Following regional workshops and meetings, the chiefdom of Engcayini (Table 3) was selected to host a pilot IDP due to the prevalence of extensive gullying, some of the worst in the country, as visible from aerial photos used by the JICA consultants. Local residents were invited to attend workshops at the planning stages of the project, convened by JICA and MOAC, where livestock were identified as the main cause of erosion on communal land due to overgrazing and the creation of cattle tracks (in combination with seasonal rains). Whilst better-off households in the chiefdom (i.e. those with cattle concerned about erosion on communal rangeland) were able to voice their concerns and preferences, the concerns of more marginalised households (i.e. households lacking cattle who were more concerned with soil fertility on arable land) were overlooked. As an outcome from the workshops, JICA and MOAC proposed some broad approaches for tackling rangeland erosion, from which the community selected what they considered to be the most appropriate intervention. However, arable farming was the main source of income for 18 % of households (2002 data), and provides subsistence for the majority of others. The desirability of a project aimed at grazing on communal rangelands as opposed to arable land thus may have been overestimated. This indicates that the framing of the project and subsequent decisions were taken without full and meaningful participation of the community.

**Table 3:** Characteristics of the study chiefdom, Engcayini

Characteristic	Detailed information
Location	Upper middleveld, approximately 30 min by car along gravel roads from Manzini, Swaziland’s largest urban settlement. Irregular and expensive transport means that access to services, markets and information are limited
Environmental characteristics	Rolling to hilly topography; slopes ranging from 15° to 30° (Jansen et al. 1994). Land is classed as good to fair in terms of production potential, with soils comprising sandy loams with patches of acid clay
Population	84 homesteads. Most households are headed by males with an average household size of 4–8 people
Power structure	Former chief has not yet been replaced, so authority lies with an acting chief who lives outside of the community. The *Indvuna* ^a^ of Engcayini lives within the chiefdom, and the village elders exert considerable authority
Livelihood activities	Waged employment, sale of arable crops, sale of natural resources, handicrafts
Arable production	All households grow maize, often in conjunction with groundnuts, sweet potatoes and beans. Drought, poor soils and parasitic weed infestations (e.g. Striga asiatica) are considered the main constraints to arable production
Livestock ownership	The number of households with cattle dropped from 68 % in 2002 to 55 % in 2014. Cattle are kept for food, draught power and manure, and are also viewed as an indicator of social status. Cattle herds have reportedly decreased in size over the last 10 years, primarily as a result of drought and disease. Goats are also kept by a number of households
Degradation problems	The topography combined with seasonal rains and thin soils in places predispose the land to erosion. Communal land is severely gullied in parts due to concentration of runoff along cattle tracks, particularly on the slopes close to the dip tank. Gullies reportedly worsened in ‘Cyclone Domonia’, which swept through the area in 1984. Soils have medium to low levels of N, P and K and widespread parasitic weed infestations indicate degraded soils in arable areas. Woodland areas supporting species used as fuel wood have decreased significantly in recent decades, but overall woodland areas have increased due to the increase of invasive species

^a^The *Indvuna* is the chairman of the local council (Inkhundla) and is selected by the chief, who may appoint any person as an *Indvuna* in respect of his chiefdom, and similarly, the chief may terminate the appointment. Should a chief be absent or a chiefdom be awaiting a new chief, the *Indvuna* may assume the role of chief, although he remains subordinate to an acting chief (Stringer et al. 2007)

#### Design

A number of schemes were designed within the project in order to tackle land degradation, including: (1) a fenced grazing scheme which subdivided the selected target communal rangeland area in order to rotate and control grazing; (2) a beef fattening feedlot scheme, which built structures with a 140 m^2^ area of concrete slabs, a water trough and a feed trough providing fodder grown in a field situated next to the feedlot; (3) a tree nursery scheme with a capacity of 160 000 seedlings was developed for agroforestry and woodlot use; (4) an afforestation scheme planted 6000 trees around a severe gully to prevent further erosion. Eucalyptus and wattle trees were mainly selected as initial tree species for the sake of soil conservation, as well as use for poles and fuel wood in future. The choice of species was JICA’s, informed by observations of successful growing and community utilisation of previous planting projects that used government resources and community labour.

#### Support

A Swazi NGO with agricultural development expertise coordinated the local activities. The NGO was expected to report on the progress of the project and to provide any necessary materials and training. It was agreed at one of the initial workshops that to ensure that the project was managed and owned as much as possible by the community, a project committee made up of representatives from the village should be democratically elected. The committee was intended to bridge the gaps between JICA, MOAC, the NGO and the community, and mobilise the community to join in with the implementation of the project. Committee members included five men and four women.

#### Implementation

Four main issues occurred during implementation. First, issues of fairness arose as the committee decided everyone should pay the same membership fee to join the scheme regardless of cattle ownership, with an additional levy on each animal that used the feedlot. Furthermore, to ensure commitment to the project, fines were imposed upon households failing to send individuals to work on the project twice a week. However, for poorer households with little or no cattle, household tasks on arable land were given priority over the rehabilitation of the rangeland, leaving them susceptible to fines. Whilst many households do not own cattle, by subscribing to the scheme they were maintaining their access rights to utilise it should they one day obtain cattle. Therefore, although seemingly unfair in the short term for those without cattle, the scheme did display elements of fairness regarding access rights in the longer term. Second, issues of capacity arose relating to the lack of awareness and capacity for participatory governance among traditional authorities, the elected project implementation committee and local residents. The community was also unaware of the full functions of the committee, and as the committee was accountable to JICA and the Swazi NGO, there was a lack of downward accountability. Third, the location and boundaries for the grazing, feedlot and nursery were situated in areas that local residents used for other purposes, such as bridleways to church, community centres, rivers, springs, forests or neighbouring households. Finally, conflicts emerged between the elected committee and traditional institutions, as the project committee began making decisions regarding the project without consulting the traditional authorities. Inadequate communication between the project committee, the elders and the acting chief were stated as the root of the problem.

#### Evaluation

Quantitative data from 2002 (Stringer et al. 2007) revealed that whilst most people were happy to work on the project (89 %): 70 % felt excluded from the project design; 57 % stated that they would have liked to be more involved in the design; and 20 % were only involved because they felt they ‘had to be’. The majority of people that felt that they gained little from the project were female, young and had no cattle. Marginalised households (i.e. poor households with little or no cattle) that could pay the standard joining fee sacrificed more in time and labour, through the opportunity cost of working on arable land, while receiving less as they had no cattle. Those with no cattle, or limited ability to pay the access fee, had potentially accessibility of land held in common reduced by the scheme. The opportunity for those with cattle to increase their wealth, coupled with the fees levied at marginalised households that lack livestock, reinforced and exacerbated pre-existing inequalities. Hence, the imposition of the project onto existing socio-political and economic structures served to consolidate the position and interests of powerful actors. Qualitative data showed that the lack of accountability resulted in many of the communities feeling that the project was owned by the committee, not the community, which generated resentment towards the committee. The legacy of exclusion, lack of awareness and capacity among authorities, and scant engagement of citizens in political processes meant that marginalised households are likely to expect those in authority to control and direct the project. This created the opportunity for elites to capture the benefits by steering the project towards more effective cattle management, while those who used the rangeland in other ways lost out and therefore placed additional pressure on the remaining communal land. Consequently, the project failed to foster meaningful community participation.

### Development of the project and the situation as of 2014

**Fig. 2:**
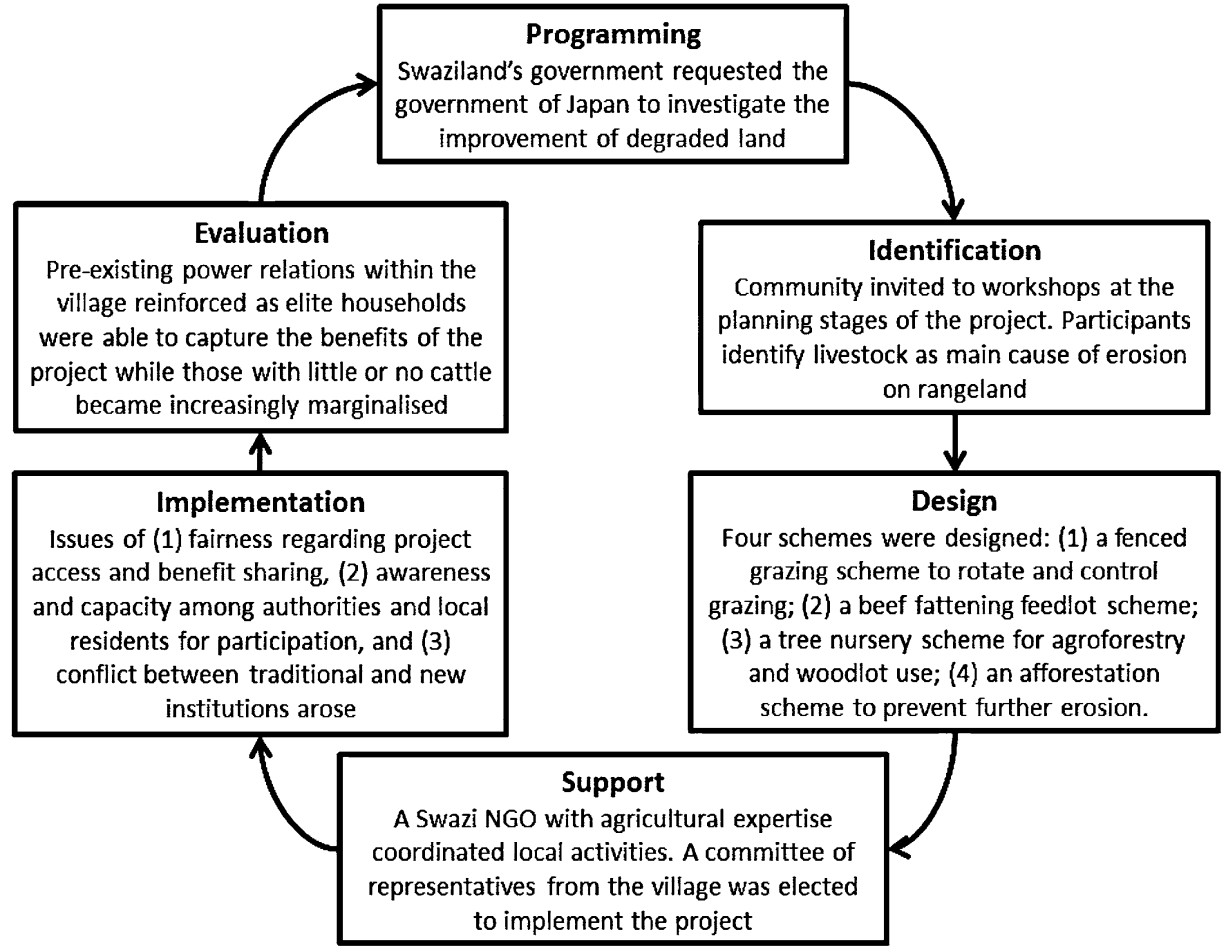
Project management cycle in relation to the JICA project

Figure 2 summarises data from 2002 together with those from 2014 within the PMC. The following sections unpack these in relation to participation, institutions, resources and outcomes.

#### Project participation

Semi-structured interviews with key households in 2014 revealed that, subsequent to feelings of exclusion during the identification and design stages of the project in 2002, respondents’ concerns about the design of the project (which they had little say in) had been vindicated as the project progressed. For example, in order to mitigate the impact of rainfall on land degradation through erosion on communal rangeland, JICA designed a system of water furrows to divert water away from a major gully in the village. This was done without community input. However, when becoming aware of the designs for the system of furrows, the villagers noted among themselves that this would not be sustainable, due to the landscape’s physical attributes and water flows that cause waterlogging in certain areas around the gully. For the villagers it was clear that the planned system of water furrows and reservoirs would eventually overflow and cause further erosion. Yet, they had no avenue to share this knowledge with JICA, who had already decided on the design of the water furrows and it was not open to debate. This demonstrates a need for deeper meaningful community participation, particularly at the stages of problem definition and design. If the community had been truly engaged in the project and been allowed the space to identify environmental priorities, perhaps some of the problems could have been avoided.

#### Project institutions

The initial project implementation committee was democratically elected to implement the project using community labour. Household interviews in 2014 supported data from 2002 that indicated friction between the committee and the traditional authorities, as well as elite capture. The *Indvuna* attempted to establish a foothold within the committee in order to take the credit for the benefits coming from the project. The chairman of the project committee challenged the actions of the *Indvuna*, arguing that they conflicted with the project objectives. However, he noted, it was very difficult to oppose the traditional authorities as they hold most power and can overrule notions from any other group. Subsequently, when the implementation phase was complete, with JICA withdrawing their support and handing over the project to the ‘community’, a new committee was appointed by the *Indvuna*. The new committee was entrusted with project maintenance, and reported directly to the *Indvuna*. However, the community felt that the lack of democratic process in the selection of the new maintenance committee meant that the best people with the right skills and motivation were not selected, rather individuals were nominated who were preferred by the *Indvuna*. This resulted in elite capture of the project.

#### Project resources

Focus groups with committee members in 2014 implied that following the withdrawal of JICA, the maintenance committee did not receive any training in project management. Furthermore, no money or other resources were available to support the maintenance of the project, contributing to the collapse of the feedlot and grazing schemes. The feedlot scheme failed partly due to lack of money for feed; the grazing scheme failed partly due to lack of funds for fertiliser and fence maintenance/replacement. Furthermore, the lack of monitoring by JICA following their withdrawal was also highlighted as a reason contributing to the failure of project schemes. The afforestation scheme was nevertheless sustained through the natural growth from the seeds of planted trees, which residents had to pay a fee to enter. Household interviews in 2014 indicate that residents felt that the failure of the project, reflected in the increasing severity of gullies, was due to poor maintenance. Residents were also dismayed at having to pay to access benefits of a project that was intended for them and implemented using their labour. Following withdrawal of JICA and appointment of the maintenance committee, the community had to pay to access any area under the project: first a fee to the *Indvuna* in order to gain a permit; and then to the committee in order to gain access to the permitted area. Failure to pay for permits resulted in the issuance of a fine. Whilst some community members were unsure where the money made from this goes, others speculated that the money is captured by the *Indvuna* and the maintenance committee. This sets up a clear structure of ‘winners’ and ‘losers’ within the community.

#### Project outcomes

Key household interviews in 2014 indicate that the community was disappointed to see the project fail and began to lose confidence in its processes. Interviewees believed this was due to the lack of motivation and commitment of the maintenance committee, noting a similar project in an adjacent village that had been more successful due to highly motivated and committed leaders. The disparity between the intended objectives of JICA and the private financial gain of local elites from the project led many residents to become disillusioned and confused as to whom the project belongs and who is responsible for it. Subsequently, whilst the community recognises the need for and potential benefit of such projects, they feel reluctant to get involved with future projects as experience shows that they primarily benefit local elites, and can even cause more problems within the community. The lack of community input and ownership, and elite capture of project benefits, resulted in loss of interest and respect for the project within the community. Many residents felt inconvenienced by the positioning of the grazing area, feedlot and nursery, and continued to use project-designated areas as bridleways. Theft and damage caused to the materials necessary for the project to successfully function (such as wire fencing, posts and gates to demarcate grazing, feedlot and woodlot areas) went virtually unchallenged.

## Discussion

### Participation

Challenges to polycentric governance, in which power is more equally distributed across levels, result from the top-down nature of IDP and lack of meaningful participation of divergent groups at various stages of the PMC. Participation is particularly challenging in contexts with traditionally top-down and highly hierarchical institutions, such as Swaziland, where communities and other groups have not traditionally had a substantial input in decision making (Stringer et al. 2009). Subsequent top-down conception and initiation of the IDP resulted in culturally inappropriate processes of community engagement and failure to align local priorities with project goals, which been shown to undermine sustainable natural resource governance (Mustalahti et al. 2012). In failing to devise adequate plans for the encouragement and sustained participation of divergent groups within the community from the outset, this constrained the ability of all groups to contribute and share their knowledge throughout its development (Andersson and Ostrom 2008). Furthermore, this resulted in misunderstanding of the project aims and objectives by local actors, and of external agents of the natural resource system and contextual factors that shape local governance (Armitage et al. 2009; Dyer et al. 2014).

Whilst local institutions strongly influence resource management (Agrawal and Benson 2011), sustainable resource management requires local participation within a collaborative framework of “cross-scale” or “multilevel” polycentric networks that go beyond local arrangements (Brondizio et al. 2009). Therefore, whilst maintaining a focus on the environmental aspects of land degradation, IDP should also provide local-level actors from diverse groups the opportunity to access institutions at multiple levels of governance. This is necessary to foster negotiation between diverse actors and knowledge systems (Nagendra and Ostrom 2012). The subsequent lack of integration of local and external actor perceptions and knowledge (see Raymond et al. 2010) failed to harness the potential of polycentric governance, i.e. to respond to environmental change by utilising the complementarities of different stakeholders and overcome the limitations and weakness of each (Andersson and Ostrom 2008). Whilst the context of Swaziland is unusual, remaining an absolute monarchy whereas most countries have moved towards constitutional monarchies, lessons here are applicable to other cases where IDPs are conceived and initiated in a top-down manner within areas where power is typically spread throughout governance levels, from local to national. It is crucial to understand such structures in order to understand the challenges to polycentric governance and devise ways to address them (Orchard et al. 2015).

### Conflict and elite capture

IDP outcomes are shaped by the pre-existing institutional structures and processes which challenge patterns of polycentric governance through institutional conflict, elite capture and ineffective, unrepresentative and opaque governance structures and processes. Establishment of the project implementation committee led to conflict between new and traditional institutions. Whilst the democratically elected committee was established to oversee implementation of the project, Tole (2010) argues that it is naïve to believe that such interventions by bureaucrats, NGOs or local leaders will be adequate for project success in heterogeneous, stratified and unequal societies. Furthermore, traditional authorities ultimately held the greatest power at the local level. Whilst initial project engagement should be conducted through traditional authorities in order to obtain their permission and approval in southern Africa, this can mean that participation at each stage of the PMC is not representative of the community (Dyer et al. 2014). Local power structures were reinforced as a result, with elites capturing project benefits owing to greater access to finance and decision-making authority, particularly as there was no corresponding opportunities for participation for checks and balances (see Mwangi and Wardell 2012).

Results indicate conflicts arising from lack of common understanding, legitimacy, monitoring and enforcement of project rules. Failure to recognise the multiscale aspects of natural resource governance obstructed polycentric governance, which has been shown to resolve conflicts from competing jurisdictions and opportunistic behaviour by elites by embracing institutional diversity and distributing power evenly between centres of power and authority (Pahl-Wostl 2009). By embracing polycentric governance, IDP can provide support for local resource users affected by land degradation to create small-scale provision networks and encourage face-to-face discussion (Biggs et al. 2012). This can foster stronger institutions that build trust and cooperation to enable local actors to devise rules for access, use, monitoring, sanctioning and resolving conflict (Mokhahlane and Obi 2011). By nesting small-scale provision networks within larger-scale networks, divergent groups could be integrated into processes susceptible to elite capture and create more responsive, transparent and accountable governance (Andersson and Ostrom 2008). This will still require a supportive central government, rather than absent or controlling, to ensure decentralisation is not appropriated by local elites (Lockwood et al. 2009).

### Outcomes

Challenges to polycentric governance throughout the PMC resulted in negative IDP outcomes of inequitable resource access, confusion and reduced trust in local institutions, and a loss of motivation to participate within future IDP in the community. In the absence of tangible benefits for marginalised households with little or no cattle, the project failed to respond to unequal benefit distribution (see Suich 2013). Johnson (2004) highlights the dangers of internal divisions that can emanate from such situations, observed when the community felt that the project was not theirs as initially intended, but instead ended up being appropriated by local elites for their own benefit. Subsequent disillusionment within the community regarding responsibilities, management procedures and enforcement mechanisms of the project resulted in the loss of legitimacy and trust in project structures and processes (see Measham and Lumbasi 2013). This supports suggestions that the political dimensions of IDP can make them vulnerable to manipulation through the marginalisation of certain stakeholder views (Khang and Moe 2008), and that the motivations and incentives of local actors, particularly elites, can shape the trajectory and outcomes of projects on the ground (see Clark et al. 2011). Therefore, caution is required as local struggles for power in the spaces of opportunity opened up by projects can be inherently destabilising and disruptive (Saunders et al. 2010).

Failure to acknowledge divergent perspectives within the community caused frustration as this leads expectations to be unfulfilled (Hogl et al. 2012). Matta and Alavalapati (2006) state that governance processes must be responsive to such negative project impacts as this shapes motivation to participate in future projects. In line with Matta and Alavalapati (2006), a lack of understanding of the divergent priorities and perspectives within the community, and lack of will or capacity for participation among those in authority, meant that governance processes were limited in their potential to respond. IDP can foster polycentric governance by creating and enabling small-scale local user networks to embed themselves within larger-scale networks of NGOs and governmental actors at multiple levels. This will facilitate effective feedback, learning and crafting of new and better solutions that can sustain community motivation to participate in natural resource management (Ostrom and Ahn 2009). By embedding such networks within polycentric governance arrangements, the locally developed but externally supported institutional arrangements are more likely to be sustained after the departure of project funders (see García-López 2013).

## Conclusion

Studying the challenges facing polycentric governance, where diversity, complexity and scale are considered to be integral components, is crucial for sustainable natural resource management (Reed and Bruyneel 2010). This paper has analysed case study data from an externally initiated IDP aiming to improve the rural environment in degraded areas of Swaziland, using a PMC framework to understand challenges to polycentric governance using empirical case study data. Using a longitudinal approach provided a useful and novel temporal dimension to improve understanding of the critical aspects of governance structures and processes that shape land use over time, and which are crucial to the study of sustainable development. This provided the opportunity to identify additional aspects that shape project success or failure that are often missed in project evaluations that take place shortly after the donor withdraws. We find a number of challenges to polycentric governance resulting from lack of participation at various stages of the PMC, namely: inappropriately defined communities and the priorities of divergent groups overlooked; conflict between centres of authority causing confusion over the aims and responsibilities of the project; pre-existing institutional structures and processes at the local level shaping inequitable benefit distributions; governance that is unresponsive to emerging challenges causing loss of community motivation for current and future IDP.

By studying the challenges to polycentric governance, through the interaction of actors between and among different levels of governance, it is possible to contribute to a more nuanced understanding of the variation in diverse governance outcomes. In order to harness the potential of polycentric governance, we recommend IDP: support communities to develop small-scale provision networks, nested within larger-scale networks of external experts, rather than the creation of committees with the sole aim of implementing externally designed plans; enable provision networks to participate in each stage of the PMC and access institutions at multiple levels of governance; facilitate collaborative institutions that embrace institutional diversity and encourage even distribution of power; sustain support of institutional linkages that build trust and cooperation for continued participation of local actors after the departure of IDP funders. Whilst the participation of local resource users at every stage of the PMC is crucial to ensure IDP are locally appropriate with community buy-in, collaboration with diverse institutions is necessary in order to harness the knowledge of various stakeholders whilst not alienating or threatening the position of traditional leaders.

Whilst a certain level of context specificity is to be expected, numerous lessons are generalizable to natural resource projects in other regions and in encouraging novel kinds of environmental management. The analysis of multi-stakeholder approaches in this context provides useful new insights to help IDP adapt participatory and collaborative models to a broader range of governance contexts, particularly where IDP are faced with powerful local actors and institutions. Future research would benefit from continued longitudinal studies to understand the dynamics of institutional arrangements for natural resource management more broadly. This would help address one of the most pivotal challenges of understanding how broader institutional contexts shape local responses and solutions to land degradation.
